# MicroRNome analysis generates a blood-based signature for endometriosis

**DOI:** 10.1038/s41598-022-07771-7

**Published:** 2022-03-08

**Authors:** Sofiane Bendifallah, Yohann Dabi, Stéphane Suisse, Ludmila Jornea, Delphine Bouteiller, Cyril Touboul, Anne Puchar, Emile Daraï

**Affiliations:** 1 Department of Obstetrics and Reproductive Medicine, Hôpital Tenon, 4 rue de la Chine, 75020 Paris, France; 2 Clinical Research Group (GRC) Paris 6: Centre Expert Endométriose (C3E), Sorbonne University (GRC6 C3E SU), Paris, France; 3 Cancer Biology and Therapeutics, Centre de Recherche Saint-Antoine (CRSA), Sorbonne University, INSERM UMR_S_938, 75020 Paris, France; 4 Sorbonne Université, Institut du Cerveau - Paris Brain Institute - ICM, Inserm, CNRS, APHP, Hôpital de la Pitié Salpêtrière, Paris, France; 5 Gentoyping and Sequencing Core Facility, iGenSeq, Institut du Cerveau et de la Moelle épinière, ICM, Hôpital Pitié-Salpêtrière, 47-83 Boulevard de l’Hôpital, 75013 Paris, France

**Keywords:** Next-generation sequencing, RNA sequencing

## Abstract

Endometriosis, characterized by endometrial-like tissue outside the uterus, is thought to affect 2–10% of women of reproductive age: representing about 190 million women worldwide. Numerous studies have evaluated the diagnostic value of blood biomarkers but with disappointing results. Thus, the gold standard for diagnosing endometriosis remains laparoscopy. We performed a prospective trial, the ENDO-miRNA study, using both Artificial Intelligence (AI) and Machine Learning (ML), to analyze the current human miRNome to differentiate between patients with and without endometriosis, and to develop a blood-based microRNA (miRNA) diagnostic signature for endometriosis. Here, we present the first blood-based diagnostic signature obtained from a combination of two robust and disruptive technologies merging the intrinsic quality of miRNAs to condense the endometriosis phenotype (and its heterogeneity) with the modeling power of AI. The most accurate signature provides a sensitivity, specificity, and Area Under the Curve (AUC) of 96.8%, 100%, and 98.4%, respectively, and is sufficiently robust and reproducible to replace the gold standard of diagnostic surgery. Such a diagnostic approach for this debilitating disorder could impact recommendations from national and international learned societies.

## Introduction

Endometriosis, characterized by endometrial-like tissue outside the uterus, is thought to affect 2–10% of women of reproductive age: representing about 190 million women worldwide^1,2^. In 2012, the World Endometriosis Research Foundation (WERF) EndoCost Consortium, including 12 tertiary care centers from 10 countries, estimated that the average cost of treating endometriosis per woman and per year amounted to 9579 € of which 3113 € were direct costs relating to care, and 6298 € indirect costs relating to loss of productivity^3^. In France, the economic burden of endometriosis management in 2017 was equivalent to that of diabetes^3^.

Early diagnosis of endometriosis is difficult as patients can present with a variety of non-specific symptoms including dysmenorrhea, dyspareunia, chronic pelvic pain, and infertility^1,2,4^: Despite the use of specific endometriosis screening questionnaires, the time from onset to diagnosis can take more than 7 years^5–8^. Moreover, a Cochrane review by Nisenblat et al. highlighted that, although imaging explorations such as transvaginal ultrasonography and magnetic resonance imaging (MRI)^9–11^ have a high accuracy in diagnosing endometrioma and some deep endometriosis locations, they exhibit poor accuracy for detecting peritoneal endometriosis which represents the early stages of the disease. Similarly, numerous studies have evaluated the diagnostic value of blood biomarkers but with disappointing results^4,12–17^. Thus, the gold standard for diagnosing endometriosis remains laparoscopy^12,13,18^.

Cumulative evidence suggests that microRNA (miRNA) dysregulation plays a pivotal role in endometriosis^4,14–19^, and several studies have investigated the potential diagnostic value of blood miRNAs^4,15,17,19^. Human miRNAs are highly conserved non-coding RNAs composed of 21–25 nucleotides which bind to their complementary messenger RNA (mRNA) thereby regulating degradation and translation of the target gene^20–23^. About 60% of genes are regulated by miRNAs^22–25^. To date, more than 2600 miRNAs have been identified in the human, but only a few hundred have been evaluated in the specific setting of endometriosis^4,17,20,22,24–26^. Some teams have attempted to build a blood-based miRNA signature to detect patients with endometriosis. Using genome-wide miRNA expression profiling by small RNA sequencing from plasma available in a biobank, Vanhie et al. identified a set of 42 miRNAs with discriminative power to differentiate between patients with and without endometriosis. Expression of 41 of these miRNAs was confirmed by RT-qPCR and three diagnostic models were built to discriminate between controls and all stages of endometriosis: minimal-mild endometriosis, and moderate to severe endometriosis. Only the model for minimal–mild endometriosis (miR-125b-5p, miR-28-5p and miR-29a-3p) exhibited an AUC of 60%, and while its sensitivity was acceptable at 78% the specificity was only 37%^14^. Selecting some miRNAs altered in endometriosis from a large screen, Moustafa et al. reported increased expression of four serum miRNAs (miR-125b-5p, miR-150-5p, miR-342-3p, miR-451a) and decreased expression of two (miR-3613-5p, let-7b). The authors concluded that their 6-miRNA signature was able to differentiate patients with endometriosis from those with other gynecologic disorders with an accuracy > 0.9^15^. However, overall, the studies in this field are based on small sample sizes limiting the validation of the signatures. Furthermore, discrepancies in methodology (study design, collection, storage, sequencing techniques, and statistical approach) have a particularly strong influence on the results of small studies^4,16,17,20,26^. In addition, miRNA selection based on the highest AUC is of low accuracy since the extreme variability of the endometriosis phenotypes has a major impact on the AUC. This may explain why signatures composed of a small selection of miRNAs are of low validity, stability, and reproducibility^4,16,17,20,26^. Thus, despite the findings of these studies, no new blood-based biomarkers are currently used in clinical practice for the diagnosis of endometriosis.

Therefore, the aim of the prospective ENDO-miRNA study, using both Artificial Intelligence (AI) and Machine Learning (ML), was to analyze the current human miRNAome to differentiate between patients with and without endometriosis, and to develop a blood-based miRNA diagnostic signature for endometriosis with internal cross-validation.

## Materials and methods

### Ethics statement

Data and plasma collection were from the prospective ENDO-miRNA study (ClinicalTrials.gov Identifier: NCT04728152). The Research Protocol (n° ID RCB: 2020-A03297-32) was approved by the ethics committee “Comité de Protection des Personnes (C.P.P.) Sud-Ouest et Outre-Mer 1” (CPP 1-20-095 ID 10476). All participants included in the study gave their written and informed consent for the use of their data. All the procedures were performed in accordance with the relevant guidelines and regulations.

The study and data analysis followed the STAndards for the Reporting of Diagnostic accuracy studies (STARD) guidelines^27^ (Annex 1). The study consisted of two parts: (i) biomarker discovery based on genome-wide miRNA expression profiling by small RNA sequencing using next generation sequencing (NGS), and (ii) development of a miRNA diagnostic signature according to expression and accuracy profiling using an ML algorithm^28–38^.

### Study population

**Figure 1:**
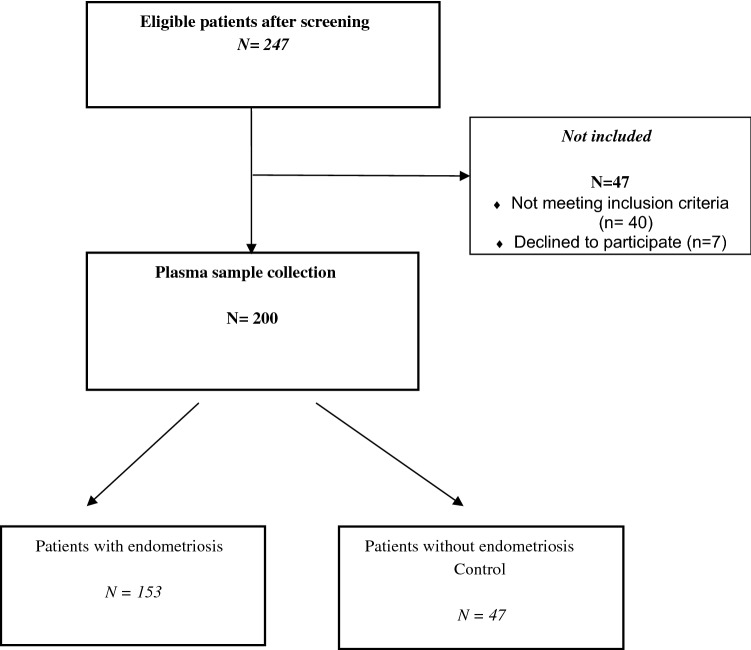
Flow chart of the ENDO-miRNA study.

The prospective ENDO-miRNA study included 200 plasma samples obtained from women with chronic pelvic pain suggestive of endometriosis. All the plasma samples were collected from the participants between January and June 2021. All the patients underwent either a laparoscopic procedure (operative or diagnostic) and/or MRI imaging^9–12^. The laparoscopic procedures were systematically videoed and then analyzed by two operators (CT, YD) who were blinded to the symptoms and imaging findings, to confirm the presence or absence of endometriosis. For the patients who underwent laparoscopy, diagnosis was confirmed by histology. Patients who were diagnosed with endometriosis without laparoscopic evaluation, all had MRI findings with features of deep endometriosis with colorectal involvement, and/or endometrioma confirmed by a multidisciplinary endometriosis committee. Following exploration by laparoscopy or MRI, the women were classified into two groups: an endometriosis group; and a control group of women with various benign pathologies other than endometriosis or with symptoms suggestive of endometriosis but without clinical or MRI features and no endometriosis lesions found during laparoscopic inspection (complex patients). The study flow chart is reported in Fig. 1. The patients with endometriosis were stratified according to the revised American Society of Reproductive Medicine (rASRM) classification^39^.

### Plasma sample collection

The blood samples (4 mL) were collected in EDTA tubes (BD, Franklin Lakes, NJ, USA) before the surgery. The plasma was isolated from whole blood within 2 h after blood sampling by two successive centrifugations at 4 °C (first at 1900*g* (3000 rpm) for 10 min, followed by 13,000–14,000*g* for 10 min to remove all cell debris), then aliquoted, labeled and stored at − 80 °C until analysis as previously described^40–42^. The miRNAs were automatically extracted with a Promega Maxwell^®^ Instrument to avoid cross contamination. Extractions and quality control (QC) were conducted in an accredited biobank (NFS96-900) to guarantee good processes. The samples were anonymized. NGS library preparation was performed individually under ISO-9001-2015 certification. QC was performed before pooling the indexed samples. After sequencing, demultiplexing was done with ILLUMINA bcl2fastq. To avoid mixing, exchanging or cross-contamination, each sample or preparation was followed with its own Laboratory Information Management System (LIMS).

### RNA sample extraction, preparation and quality control

RNA was extracted automatically from 500 μL of plasma using a Maxwell 48^®^ RSC Instrument together with the Maxwell^®^ RSC miRNA Plasma and Serum Kit (ref AS1680, Promega, USA) according to the manufacturer’s protocol. Libraries for small RNA sequencing were prepared using the QIAseq miRNA Library Kit for Illumina (Qiagen, Germany). The resulting small RNA libraries were concentrated by ethanol precipitation and quantified using a Qubit 2.0 Fluorometer (Thermo Fisher Scientific, USA) prior to sequencing on a Novaseq 6000 sequencer (Illumina, USA) with read lengths of 100 bases and 17 million single-end reads per sample, on average^43–45^.

### Bioinformatics

#### Raw data preprocessing (raw, filtered, aligned reads) and quality control

Sequencing reads were processed using the data processing pipeline. FastQ files were trimmed to remove adapter sequences using Cutadapt version v.1.18 and were aligned using Bowtie version 1.1.1 to the following transcriptome databases: the human reference genome available from NCBI (https://www.ncbi.nlm.nih.gov/genome/guide/human/), and miRbase (v22) (miRNAs) using the MirDeep2 v0.1.0 package. The raw sequencing data quality was assessed using FastQC software v0.11.7^46^.

#### Differential expression analysis of miRNA

Expression level quantification of the miRNAs was first determined by miRDeep2^47^. Differential expression tests were then conducted in DESeq2 only for the miRNAs with read counts in ≥ 1 of the samples. DESeq2 integrates methodological advances with several novel features to facilitate a more quantitative analysis of comparative RNA-seq data using shrinkage estimators for dispersion and fold change^48,49^. miRNAs were considered as differentially expressed if the absolute value of log2-fold change was > 1.5 (upregulated) and < 0.5 (downregulated). The P value adjusted for multiple testing was < 0.05^48^.

### Statistical analysis

#### Feature selection

In the present study, we mixed 10 different methods to score all the miRNAs present in the 200 sequencing samples. For each method, we estimated the importance of each miRNA and retained the top scoring miRNAs.

#### Development and internal validation of the diagnostic model

ML was trained to develop a diagnostic signature for endometriosis. ML models such as Logistic Regression (LR), Random Forest (RF), eXtreme Gradient Boosting (XGBoost), and AdaBoost are considered ensemble learning techniques^28–31,50–52^. To assess and compare the diagnostic performance of the diagnostic signature, the sensitivity, specificity, and Receiver Operating Characteristics (ROC) Area Under the Curve (AUC) were calculated^53,54^. The signature accuracy and reproducibility for each ML model were internally cross validated on 10 random data sets composed of the identical proportion of control and endometriosis patients. ML analysis was performed using Python (Python Software Foundation) with scikit-learn 0.19.1, xgboost 1.3.3, and scipy 1.1 packages.

### Other statistical analyses

Statistical analysis was based on the Chi^2^ test as appropriate for categorical variables. Values of P < 0.05 were considered to denote significant differences. Data were managed with an Excel database (Microsoft, Redmond, WA) and analyzed using R 2.15 software, available online (https://www.r-project.org/).

## Results

### Description of the ENDO-miRNA cohort

The ENDO-miRNA study included 200 patients, with 76.5% (n = 153) who were diagnosed with endometriosis, and 23.5% (n = 47) without (controls), respectively. Among patients with endometriosis, 52% (n = 80) and 48% (n = 73) were staged rASRM stage I–II versus with III-IV. The control group is composed in majority (51% (n = 24)) by women with no abnormality after laparoscopic diagnostic. The clinical and demographics characteristics of patients are summarized in Table 1.

**Table 1:** Demographic Characteristics of the population.

	Controls N (%) N = 47	Endometriosis N (%) N = 153	
Age (mean ± SD)	30.92 (13.79)	31.17 (10.78)	0.1912
BMI (body mass index) (mean ± SD)	24.84 (11.10)	24.36 (8.38)	0.525
**rASRM classification**			–
I–II	–	52% (80)	
III–IV	–	48% (73)	
**Control diagnoses**			
No abnormality	51% (24)	–	–
Leiomyoma	2% (1)		
Cystadenoma	11% (5)		
Teratoma	23% (11)		
Others gynecological disorders	13% (6)		
Dysmenorrhea	100%	100%	
**Abdominal pain outside menstruation**			
Yes	66% (21)	71% (89)	0.6905
**Pain suggesting sciatica**			
Yes	31% (10)	56% (70)	0.0214
Dyspareunia (mean ± SD)	4.95 (3.52)	5.28 (3.95)	< 0.001
**Lower back pain outside menstruation**			
Yes	62% (20)	81% (101)	0.0498
Painful defecation (mean ± SD)	2.84 (2.76)	4.35 (3.47)	< 0.001
**Right shoulder pain during menstruation**			
Yes	9% (3)	21% (26)	0.2184
Urinary pain during menstruation (mean ± SD)	2.84 (2.76)	4.35(3.36)	< 0.001
**Blood in the stools during menstruation**			
Yes	12% (4)	24% (30)	0.2425
**Blood in urine during menstruation**			
Yes	25% (8)	17% (21)	0.4172

There were no significant differences in terms of age and body mass index (BMI) between the groups. Compared to the control group, the endometriosis group had higher rates of sciatica pain (p = 0.021), dyspareunia (p < 0.001), lower back pain outside menstruation (p = 0.049), and urinary pain during menstruation (p < 0.001).

### Global overview of the miRNA transcriptome

The sequencing of the 200 plasma samples for small RNA-seq provided ~ 4228 M raw sequencing reads (from ~ 11.7 M to ~ 34.98 M reads/sample). After filtering steps, we retained 39% (~ 1639 M) of initial raw reads. Among those, the majority of were described as 20–23 nt length which corresponds to mature miRNA sequences. The identification of known miRNAs provided ~ 2588 M sequences which have been mapped to 2633 known miRNAs from miRbase (v22). The expressed miRNAs ranged from 666 to 1274 per blood sample. The overall composition of processed reads is shown in Annex 2.

### Accuracy of the miRNAs to diagnose endometriosis

Of the 2561 miRNAs known to be related to endometriosis, the feature selection generated a subset of 86 miRNAs. According to the F1-score, sensitivity, specificity and AUC values ranged from 0–88.2%, 0–99.4%, 4–100%, and 50–68%, respectively. Among the 86 miRNAs selected, 20% (n = 69) had an AUC value < 60%, and 80% (n = 17) a value ≥ 60%; for the FI-scores, 50% (n = 43) and 50% (n = 43) had a value ranging between 0–79%, and ≥ 80%, respectively; 51% (n = 44) and 49% (n = 42) had a sensitivity ranging between 0–79%, and ≥ 80%, respectively; and 77% (n = 94) and 23% (n = 20) had a specificity ranging between 0–79%, and ≥ 80%, respectively. Among these, 42% (n = 36) were identified as being downregulated, 6% (n = 5) as being upregulated, and 52% (n = 45) as being unregulated. Annex 3 summarizes the relative expression of a panel of the most accurate miRNAs for dysmenorrhea, hormonal treatment status, and rASRM stage (I–II vs III–IV). The signature composition and a summary of the diagnostic accuracy of each of the 86 miRNAs selected is reported in Table 2.

**Table 2:** miRNAs accuracies for diagnose endometriosis.

miR	AUC	F1-score	Sensibility	Specificity	Regulation
miR-3622a-3p	0.5	0	0	1	DOWN
miR-504-3p	0.567	0.871	0.961	0.174	DOWN
miR-526a-3p	0.548	0.876	0.987	0.109	–
miR-124-3p	0.656	0.796	0.747	0.565	–
miR-3923	0.557	0.868	0.961	0.152	DOWN
miR-5004-3p	0.562	0.882	0.994	0.13	DOWN
miR-520h	0.537	0.874	0.987	0.087	–
miR-5700	0.5	0	0	1	–
miR-6502-5p	0.603	0.443	0.292	0.913	UP
miR-6799-3p	0.564	0.867	0.955	0.174	DOWN
miR-6826-5p	0.588	0.776	0.74	0.435	–
miR-6837-5p	0.56	0.871	0.968	0.152	–
miR-7108-3p	0.594	0.816	0.818	0.37	DOWN
miR-1180-5p	0.564	0.867	0.955	0.174	DOWN
miR-3064-3p	0.574	0.851	0.909	0.239	DOWN
miR-3168	0.618	0.803	0.779	0.457	DOWN
miR-3185	0.566	0.878	0.981	0.152	DOWN
miR-4674	0.57	0.797	0.792	0.348	DOWN
miR-4764-5p	0.529	0.874	0.994	0.065	DOWN
miR-516a-3p	0.5	0	0	1	–
miR-542-5p	0.619	0.854	0.89	0.348	–
miR-889-5p	0.563	0.875	0.974	0.152	DOWN
miR-1253	0.578	0.847	0.896	0.261	DOWN
miR-1292-5p	0.61	0.771	0.721	0.5	–
miR-138-1-3p	0.599	0.661	0.545	0.652	–
miR-1910-5p	0.555	0.875	0.981	0.13	DOWN
miR-216b-3p	0.551	0.879	0.994	0.109	DOWN
miR-26a-2-3p	0.594	0.532	0.383	0.804	–
miR-29b-1-5p	0.68	0.781	0.708	0.652	UP
miR-30e-3p	0.627	0.579	0.429	0.826	–
miR-3117-5p	0.5	0	0	1	–
miR-3122	0.59	0.333	0.201	0.978	UP
miR-3137	0.617	0.779	0.734	0.5	DOWN
miR-4696	0.5	0	0	1	–
miR-4703-5p	0.551	0.879	0.994	0.109	DOWN
miR-4715-5p	0.587	0.324	0.195	0.978	UP
miR-4740-5p	0.5	0	0	1	DOWN
miR-4749-5p	0.58	0.777	0.747	0.413	DOWN
miR-4797-3p	0.578	0.643	0.526	0.63	–
miR-4804-5p	0.596	0.764	0.714	0.478	–
miR-4999-5p	0.612	0.637	0.506	0.717	–
miR-5681a	0.5	0	0	1	–
miR-6075	0.562	0.856	0.929	0.196	DOWN
miR-6509-5p	0.606	0.777	0.734	0.478	–
miR-6824-3p	0.552	0.872	0.974	0.13	DOWN
miR-6875-3p	0.553	0.865	0.955	0.152	DOWN
miR-1278	0.612	0.761	0.701	0.522	–
miR-1343-5p	0.611	0.826	0.831	0.391	–
miR-1973	0.529	0.874	0.994	0.065	DOWN
miR-203a-5p	0.5	0	0	1	DOWN
miR-208a-3p	0.579	0.818	0.831	0.326	DOWN
miR-208a-5p	0.569	0.863	0.942	0.196	DOWN
miR-3124-5p	0.604	0.491	0.338	0.87	–
miR-3176	0.596	0.764	0.714	0.478	–
miR-3683	0.568	0.784	0.766	0.37	–
miR-3691-5p	0.599	0.561	0.416	0.783	–
miR-375-5p	0.529	0.874	0.994	0.065	–
miR-3939	0.558	0.303	0.182	0.935	–
miR-3975	0.5	0	0	1	–
miR-4260	0.5	0	0	1	–
miR-4295	0.518	0.872	0.994	0.043	–
miR-4296	0.529	0.874	0.994	0.065	–
miR-433-3p	0.605	0.672	0.558	0.652	–
miR-4445-3p	0.518	0.872	0.994	0.043	–
miR-4455	0.5	0	0	1	–
miR-4511	0.624	0.753	0.682	0.565	–
miR-4536-3p	0.549	0.178	0.097	1	UP
miR-4655-5p	0.604	0.59	0.448	0.761	–
miR-4725-5p	0.537	0.874	0.987	0.087	DOWN
miR-4738-5p	0.567	0.76	0.721	0.413	–
miR-4750-3p	0.529	0.874	0.994	0.065	DOWN
miR-514b-5p	0.555	0.875	0.981	0.13	DOWN
miR-548aw	0.578	0.54	0.396	0.761	–
miR-548w	0.584	0.569	0.429	0.739	–
miR-5572	0.537	0.874	0.987	0.087	–
miR-5702	0.534	0.87	0.981	0.087	–
miR-573	0.54	0.851	0.929	0.152	DOWN
miR-6788-3p	0.545	0.873	0.981	0.109	DOWN
miR-6811-3p	0.549	0.869	0.968	0.13	DOWN
miR-6813-5p	0.622	0.763	0.701	0.543	–
miR-6830-5p	0.542	0.862	0.955	0.13	–
miR-6872-3p	0.518	0.872	0.994	0.043	–
miR-6888-5p	0.529	0.874	0.994	0.065	–
miR-7109-5p	0.549	0.869	0.968	0.13	DOWN
miR-7150	0.57	0.855	0.922	0.217	DOWN
miR-7152-5p	0.5	0	0	1	DOWN

### Diagnostic importance of the miRNAs for blood signature

**Figure 2:**
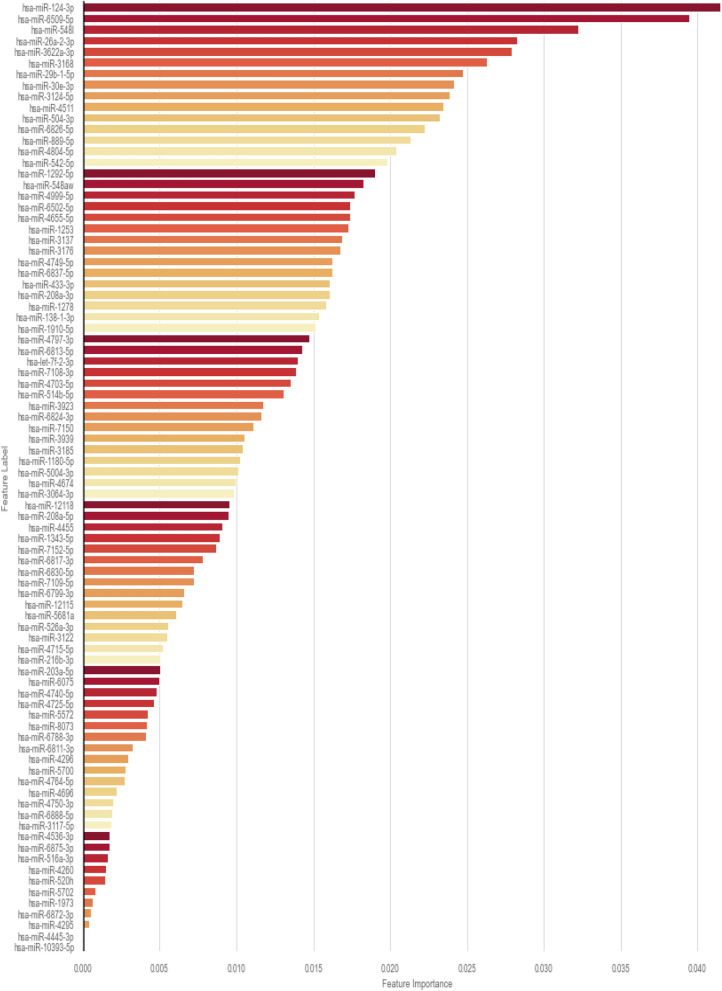
Relative importance of each miRNA in the final signature.

Among the 86 miRNAs composing the blood signature, 10 have the greatest potential value: namely, miRNAs 124-3p, 6509-5p, 548l, 26a-2-3p, 3622a-3p, 3168, 29b-1-5p, 30e-3p, 3124-5p, 4511. The diagnostic importance of the miRNAs is reported in Fig. 2. Among these 10 miRNAs, one (miRNA124-3p) has been previously reported in the setting of endometriosis.

### miRNA blood-based diagnostic signature for endometriosis

The overall performance of the ML models against the 10 datasets are reported in Table 3. Against the 10 datasets randomly generated, the sensitivity, specificity, and AUC ranged from 80.6 to 96.8%, 77.8 to 100%, and 76.2 to 98.4%, respectively. The most accurate signature (n°3) after internal cross-validation provides a sensitivity, specificity, and AUC of 96.8%, 100%, and 98.4%, respectively (Table 3).

**Table 3:** Comparison of ML model accuracy to diagnose endometriosis.

Datasets	Random Forest			XGBoost			AdaBoost			Logistic regression		
	AUC	Sensitivity	Specificity	AUC	Sensitivity	Specificity	AUC	Sensitivity	Specificity	AUC	Sensitivity	Specificity
1	0.935	0.871	1	0.952	0.903	1	0.935	0.871	1	0.887	0.774	1
2	0.984	0.968	1	0.984	0.968	1	0.984	0.968	1	0.968	0.935	1
3	0.984	0.968	1	0.952	0.903	1	0.984	0.968	1	0.984	0.968	1
4	0.912	0.935	0.889	0.896	0.903	0.889	0.912	0.935	0.889	0.919	0.839	1
5	0.967	0.933	1	0.967	0.933	1	0.9	0.9	0.9	0.933	0.867	1
6	0.896	0.903	0.889	0.896	0.903	0.889	0.88	0.871	0.889	0.912	0.935	0.889
7	0.984	0.968	1	0.984	0.968	1	0.984	0.968	1	0.984	0.968	1
8	0.952	0.903	1	0.968	0.935	1	0.935	0.871	1	0.919	0.839	1
9	0.968	0.935	1	0.968	0.935	1	0.935	0.871	1	0.864	0.839	0.889
10	0.983	0.967	1	0.967	0.933	1	0.95	0.9	1	0.883	0.767	1

### Relation between pathophysiology of endometriosis and miRNA expression

**Figure 3:**
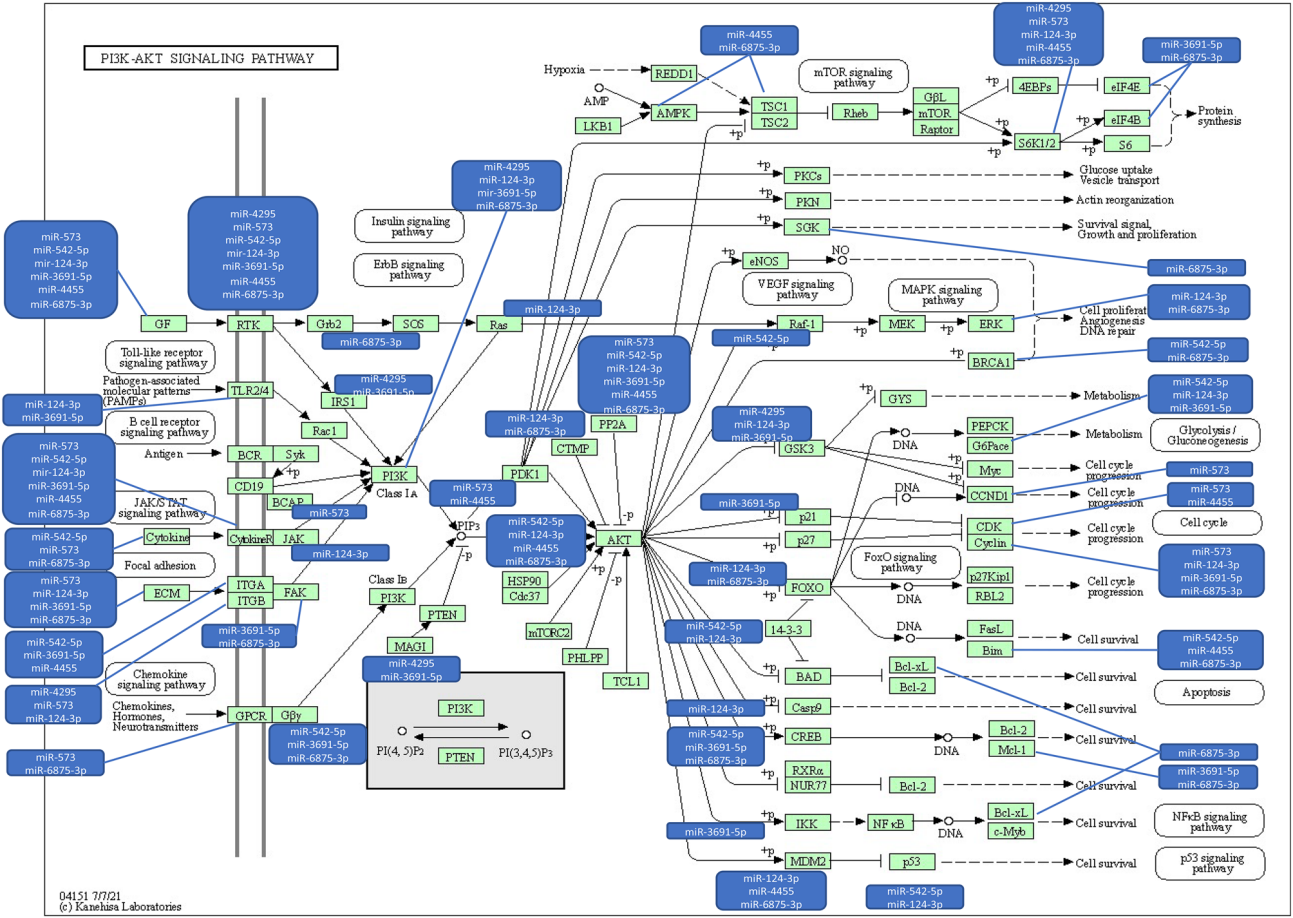
Network, pathways, and functions for the relevant miRNAs associated with PI3K/Akt, MAPK pathways (with the Copyright permission of KEGG https://www.kegg.jp/kegg/kegg1.html with the reference number Ref: 220,170).

Among the 86 miRNAs composing the diagnostic signature, 40.7% (35/86) have not been previously described in the human. The remaining have been described in both benign and malignant conditions (Table 4). Almost 30% of the 86 miRNAs are downregulated, and many of them are related to the PI3K/Akt and MAPK pathways. Figure 3 illustrates the network, pathways, and functions for the relevant miRNAs associated with these pathways^55,56^. Only miR-124-3p has previously been reported in patients with endometriosis. Details concerning the exhaustive signaling pathways and targeted regulators are summarized in Annex 4.

**Table 4:** Disorders previously associated with the miRNAs of the endometriosis—signature.

miRNAs	Previously described	Endometriosis field	Benign disorder	Malignant disorder
hsa-miR-3622a-3p	Yes	No	–	Colorectal cancer
hsa-miR-504-3p	Yes	No	Marker of nonalcoholic fatty liver disease	
hsa-miR-124-3p	Yes	Yes	Peripheral arterial disease, Hypertension, acute respiratory distress syndrom, Parkinson	Ovarian cancer, HCC, Gastric cancer, Glioma, breast cancer
hsa-miR-3923	Yes	No		Pancreatic cancer, Predict metastasis in breast cancer
hsa-miR-5004-3p	Yes	No	SARS Cov 2	
hsa-miR-520h	Yes	No	Diabetic nephropathy	Breast cancer, Colorectal cancer, Renal cancer
hsa-miR-6826-5p	Yes	No		Cervical cancer
hsa-miR-1180-5p	Yes	No		Wilm's tumor, Bladder cancer
hsa-miR-3168	Yes	No		HCC
hsa-miR-3185	Yes	No		Associated with death by mechanical asphyxia, CHC
hsa-miR-4674	Yes	No	Alzheimer disease	Associated with distant metastasis in prostatic cancer
hsa-miR-4764-5p	Yes	No	Associated with Rhumatoid arthritis	
hsa-miR-516a-3p	Yes	No		Breast cancer, cirrhosis, gastric cancer
hsa-miR-542-5p	Yes	No	Diabetic retinopathy, myocardial injury	Osteosarcoma, breast cancer, gastric cancer, colorectal cancer
hsa-miR-1253	Yes	No	Vascular Smooth muscle, Hypertension complications,	Medulloblastoma, NSCLC, HCC
hsa-miR-1292-5p	Yes	No		Gastric cancer
hsa-miR-138-1-3p	Yes	No		Nasopharyneal carcinoma, Lung cancer, thyroid cancer, Renal cancer
hsa-miR-1910-5p	Yes	No	Associated with response to oxydative stress	
hsa-miR-216b-3p	Yes	No		Lung cancer, Pancreatic cancer
hsa-miR-29b-1-5p	Yes	No	Helicobacter Pylori, ischemia, cardiomyocytes, endometrium repair	Triple negative breast cancer, colon cancer, oral squamous cell carcinoma, bladder cancer
hsa-miR-30e-3p	Yes	No	Nervous system, Cardiomyocytes	Glioma, Hepatocellular carcinoma, ovarian cancer, renal carcinoma
hsa-miR-3122	Yes	No		Functional polymorphisms associated with breast cancer susceptibility in Chinese Han population
hsa-miR-4703-5p	Yes	No		Pancreatic cancer
hsa-miR-4715-5p	Yes	No		Lung cancer
hsa-miR-4749-5p	Yes	No		Glioblastoma
hsa-miR-4999-5p	Yes	No		Colorectal
hsa-miR-6075	Yes	No		Pancreatic and biliary tract cancers, lung cancers
hsa-miR-6509-5p	Yes	No		Hepatocellular carcinoma
hsa-miR-6875-3p	Yes	No		Hepatocellular carcinoma
hsa-miR-1278	Yes	No		Papillary cancer, lung cancer, ovarian cancer
hsa-miR-1973	Yes	No	Spermatogenic impairments, biomarker for detecting T21	Prostate cancer, Hodgkin lymphoma, early colon carcinoma, renal cancer
hsa-miR-203a-5p	Yes	No	Peridontis, foot and mouth virus	Cervical cancer, lung cancer, oropharyngeal cancer
hsa-miR-208a-3p	Yes	No	Acute myocardial infarction and cardiac remodeling	Colorectal cancer, osteosarcoma
hsa-miR-208a-5p	Yes	No		Bladder cancer
hsa-miR-3691-5p	Yes	No		Hepatocellular cancer, lung cancer
hsa-miR-375-5p	Yes	No	Marker of Diabetes type 1	Teratoma in testicular cancer
hsa-miR-3939	Yes	No	Diabetic retinopathy and type 2 diabetes metillus	
hsa-miR-4260	Yes	No		Acute myeloid leukemia, colorectal cancer
hsa-miR-4295	Yes	No	Hemangioma	Osteosarcoma, head and neck carcinoma, bladder cancer, glioma, gastric cancer, ductal pancreatic carcinoma, Non small cells lung cancer
hsa-miR-4296	Yes	No		Osteosarcoma
hsa-miR-4455	Yes	No		Gastric cancer
hsa-miR-4536-3p	Yes	No		Non-small cell lung cancer
hsa-miR-4750-3p	Yes	No		Pancreatic cancer
hsa-miR-514b-5p	Yes	No		Colorectal cancer
hsa-miR-5572	Yes	No	Sporadic amyotrophic lateral sclerosis	
hsa-miR-5702	Yes	No		Non-small cell lung cancer, triple negative breast cancer
hsa-miR-573	Yes	No	Intervertebral disc degeneration, rheumatoid arthritis	Pancreatic cancer, prostate cancer, hepatocellular carcinoma, BRCA1—Mediated breast cancers, Melanoma, gastric cancer, cervical cancer, lung cancer
hsa-miR-6813-5p	Yes	No		Breast cancer
hsa-miR-6872-3p	Yes	No	Human cartilage	
hsa-miR-7109-5p	Yes	No		Oral squamous cell carcinoma
hsa-miR-7150	Yes	No	Human cartilage	–

## Discussion

We present here a blood-based diagnostic signature combining a selected panel of 86 miRNAs extracted from patients with chronic pelvic pain suggestive of endometriosis participating in the prosspective ENDO-miRNA study.

To the best of our knowledge, this is the first blood-based diagnostic signature obtained from a combination of two robust and disruptive technologies merging the intrinsic quality of miRNAs to condense the endometriosis phenotype (and its heterogeneity) with the modeling power of AI. The most accurate signature provides a sensitivity, specificity, and AUC of 96.8%, 100%, and 98.4%, respectively, and is sufficiently robust and reproducible to replace the gold standard of diagnostic surgery.

We hypothesize that this signature could have large implications for clinical practice in improving endometriosis care pathways by significantly reducing time to diagnosis and therapeutic wandering.

In the specific setting of endometriosis, multiple biomarkers^13,18,64^, genomic analyses^32,57^, questionnaires^5,58,59^, symptom-based algorithms^5^, and imaging techniques^12^ have been advocated as screening and triage tests for endometriosis. However, to date, none have demonstrated sufficient clinical accuracy, i.e., a sensitivity of 0.94 and specificity of 0.79^12,13,18^. The present signature composed of 86 miRNAs exceeds the required sensitivity and specificity metrics suggesting high clinical value. In addition, as stated by Agrawal et al.^4^ the main characteristic’s for relevant biomarker for clinical use is one which is (i) specific to the disorder, (ii) associated with early stage of the disease, (iii) accessible and acceptable with non-invasive procedure, (iv) biologically stable and clinically reproducible, and (v) associated with known or potential pathophysiological mechanisms. Therefore, to subscribe to Agrawal et al.’s criteria and improving endometriosis diagnosis, the prospective ENDO-miRNA study was designed to analyze the entire humain miRNome especially for (i) complex women (women with chronic pelvic pain suggestive of endometriosis and both negative clinical examination and imaging findings), (ii) women various phenotypes based on early and advanced stages (I–II vs III–IV rASRM) and (v) women with other gynecologic disorders sharing the symptoms of endometriosis. The exhaustive analyze of all miRNAs (n = 2633) from 200 blood samples of patients with without endometriosis allow to capture the complexity of the disease and in fine to illustrate its heterogeneity. The data that emerged from this analysis, resulted in the combination of a large set of 86 miRNAs robustly selected by 10 reproducible statistical methods (and not only based on the AUC criteria as previous reports). miRNA selection based purely on the highest AUC is of low accuracy because the extreme variability of endometriosis has a major impact on AUC. This point may explain the low validity, stability and reproducibility of using a few miRNAs to design a signature.

To date, only studies evaluating a limited number of mi-RNAs^14,17,20,21,26^ using classic logistic regression have been published. These studies show that some miRNAs are deregulated in patients with endometriosis. For example, in a retrospective study using blood samples from a biobank, Vanhie et al.^14^ failed to build a signature based on 42 miRNAs divided into three models of three miRNAs each, mainly because the authors focused on the accuracy of each miRNA to design a signature. In agreement with Lopez-Rincon et al.^36–38^ it would appear illusory that endometriosis—a highly heterogeneous multifactorial disorder with various phenotypes and characterized by incomplete knowledge of the various pathologic pathways—could be reflected by a few miRNAs. Therefore, we decided (i) to select specific miRNAs based on 10 statistical methods (resulting in a selection of 86 miRNAs), and (ii) to use several highly accurate ML models which support the value of AI technology as a disruptive approach. Such an approach has been previously validated in cancer showing that a 100-miRNA signature was sufficiently stable to provide almost the same classification accuracy across different types of cancers and platforms^36,37^.

Numerous studies have evaluated blood or plasma miRNA expression as potential biomarkers for endometriosis but with discordant results, probably because of study design issues but also because of limitations inherent to the biological techniques used^17^. For example, Yang et al.^60^ found 61 miRNAs (36 downregulated and 25 upregulated) significantly expressed in the serum of patients with endometriosis by array analysis, but only five were validated by qRT-PCR. These data underline the importance of NGS platforms for miRNA profiling. Although considerable computational support is needed, these platforms are of high sensitivity and resolution, and of excellent reproducibility allowing the analysis of millions of RNA fragments. As described by A C ‘t Hoen et al.^61^, bioinformatics allows the exhaustive analysis of all RNA fragments that can be aligned and mapped, and their expression levels quantified, thus eliminating the need for sequence specific hybridization probes or qRT-PCR which are required in a microarray^62^.

From a pathophysiologic point of view, a systematic review revealed that 45% of the 86 miRNAs composing our endometriosis signature have not previously been reported in the human. Only miR-124-3p has previously been reported in patients with endometriosis, and is involved in ectopic endometrial cell proliferation and invasion in both benign and malignant disorders^63^. In addition, miR-124-3p has been found to be involved in various signaling pathways such as mTOR STAT3, PI3K/Akt, NF-κB, ERK, PLGF-ROS, FGF2-FGFR, MAPK, GSK3B/$\beta$-catenin^64,65^. The remaining miRNAs of the signature have previously been identified as being involved in both benign and malignant disorders with the main signaling pathways being JAK/STAT, NF-KB, YAP/TAZ, PIK3/Akt, Wnt/$\beta$-catenin, FOXO, MAPK, p53, mTOR and TGF-ß. All these data open new avenues to better understand the pathophysiology of endometriosis and to develop new therapeutic options already used in other pathologies.

Some limits of the present study deserve to be discussed. First, some of our patients—in both the endometriosis and control group—had a prior hormonal treatment that may have affected miRNA expression. However, Vanhie et al. reported that no miRNAs changed significantly with the menstrual cycle^14^. Moreover, Moustafa et al. found that miRNAs remained unchanged both throughout the menstrual cycle and in response to sex steroid hormone treatment^15^. Second, among the 10 miRNAs with the most important diagnostic value only miRNA124-3p has been previously reported in the setting of endometriosis which suggests that external validation is required. Third, our signature was based on patients aged between 18 and 43 years excluding adolescents with pelvic pain. Therefore, an additional study should be performed for adolescent patients. Fourth, although no difference was observed in miRNA expression between patients with dysmenorrhea under or over VAS 7, no attempt was made to correlate symptoms with the various locations of endometriosis. Finally, some patients with deep endometriosis and/or endometrioma were included in the endometriosis group without having undergone laparoscopy and this represents a potential bias. However, the meta-analysis by Nisenblat et al. demonstrated that MRI fulfills the criteria for a replacement and SnNout triage test for endometrioma, colorectal and pouch of Douglas obliteration related to endometriosis^12^.

## Conclusion and perspectives

The present study supports the use of a blood-based miRNA signature of endometriosis. Such a diagnostic approach for this debilitating disorder could impact recommendations from national and international learned societies. Beyond the diagnostic value of our endometriosis signature, the combined methodology using AI and ML could better determine the prognosis and natural history of the various phenotypes of the disease, and evaluate the response to medical and surgical treatments, especially in infertile patients. On a broader scale, the current methodology is also suitable as a model for other multifactorial benign disorders as well as for cancer.

## Supplementary Information

Supplementary Information 1.

Supplementary Information 2.

Supplementary Information 3.

Supplementary Information 4.

Supplementary Information 5.
