# Supplementary figures and images for: MicroRNome analysis generates a blood-based signature for endometriosis
Source: Sci Rep. 2022 Mar 8;12:4051. doi: 10.1038/s41598-022-07771-7 (PMC8902281; doi:10.1038/s41598-022-07771-7)

**Annex 2.** Overall composition of processed reads


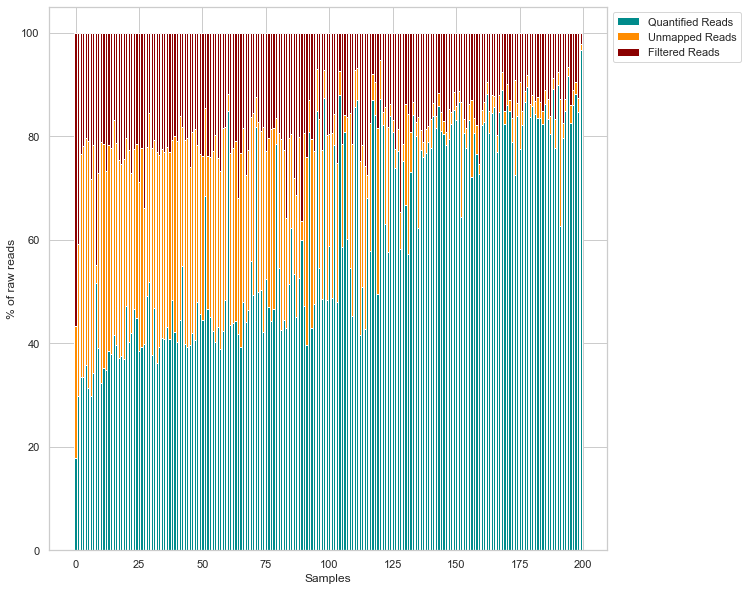

Supplement: Supplementary file 2 — Supplementary Information 2. [file 41598_2022_7771_MOESM2_ESM.docx]
