# Supplementary material for: MicroRNome analysis generates a blood-based signature for endometriosis
Source: Sci Rep. 2022 Mar 8;12:4051. doi: 10.1038/s41598-022-07771-7 (PMC8902281; doi:10.1038/s41598-022-07771-7)
Supplement: Supplementary file 3 — Supplementary Information 3. [file 41598_2022_7771_MOESM3_ESM.docx]

Annex 3 :

1. Panel of 5 miRNAs profile expression according to dysmenorrhea level

b) Panel of 5 miRNA profile expression according to rASRM stage

c) Panel of 5 miRNA profile expression according to hormonal treatment use
