# Supplementary material for: MicroRNome analysis generates a blood-based signature for endometriosis
Source: Sci Rep. 2022 Mar 8;12:4051. doi: 10.1038/s41598-022-07771-7 (PMC8902281; doi:10.1038/s41598-022-07771-7)
Supplement: Supplementary file 4 — Supplementary Information 4. [file 41598_2022_7771_MOESM4_ESM.docx]

**Annex summary**

**Annex 1.** Standards for Reporting of Diagnostic Accuracy (STARD) check list

**Annex 2.** Overall composition of processed reads

**Annex 3.** miRNA expression according to dysmenorrhea, hormonal treatment status, and to rASRM stage (I-II vs III-IV).

**Annex 4.** The details of exhaustive signaling pathways and targeted regulators for the 86 miRNAs of the signature
